# Gray Matter Changes in Adolescents Participating in a Meditation Training

**DOI:** 10.3389/fnhum.2020.00319

**Published:** 2020-08-14

**Authors:** Justin P. Yuan, Colm G. Connolly, Eva Henje, Leo P. Sugrue, Tony T. Yang, Duan Xu, Olga Tymofiyeva

**Affiliations:** ^1^Department of Radiology & Biomedical Imaging, University of California, San Francisco, San Francisco, CA, United States; ^2^Department of Psychology, Stanford University, Stanford, CA, United States; ^3^Department of Biomedical Sciences, Florida State University College of Medicine, Tallahassee, FL, United States; ^4^Department of Psychiatry and Behavioral Sciences, The Langley Porter Psychiatric Institute, Division of Child and Adolescent Psychiatry, Weill Institute for Neurosciences, University of California, San Francisco, San Francisco, CA, United States; ^5^Department of Clinical Science/Child and Adolescent Psychiatry, Umeå University, Umeå, Sweden

**Keywords:** meditation, adolescent brain, gray matter, voxel-based morphometry, MRI

## Abstract

Meditation has shown to benefit a wide range of conditions and symptoms, but the neural mechanisms underlying the practice remain unclear. Magnetic resonance imaging (MRI) studies have investigated the structural brain changes due to the practice by examining volume, density, or cortical thickness changes. However, these studies have focused on adults; meditation’s structural effects on the adolescent brain remain understudied. In this study, we investigated how meditation training affects the structure of the adolescent brain by scanning a group of 38 adolescents (16.48 ± 1.29 years) before and after participating in a 12-week meditation training. Subjects underwent Training for Awareness, Resilience, and Action (TARA), a program that mainly incorporates elements from mindfulness meditation and yoga-based practices. A subset of the adolescents also received an additional control scan 12 weeks before TARA. We conducted voxel-based morphometry (VBM) to assess gray matter volume changes pre- to post-training and during the control period. Subjects showed significant gray matter (GM) volume decreases in the left posterior insula and to a lesser extent in the left thalamus and left putamen after meditation training. There were no significant changes during the control period. Our results support previous findings that meditation affects regions associated with physical and emotional awareness. However, our results are different from previous morphometric studies in which meditation was associated with structural increases. We posit that this discrepancy may be due to the differences between the adolescent brain and the adult brain.

## Introduction

Meditation is a mind-body practice that has become increasingly popular both recreationally and clinically (Goyal et al., 2014). It can be defined as a form of mental training that aims to improve one’s psychological capabilities and it encompasses multiple practices, most notably mindfulness meditation (Ospina et al., 2008; Tang et al., 2015). Overall, meditation’s extensive health benefits are well-documented. In addition to reducing stress (Grossman et al., 2004), mindfulness meditation has demonstrated efficacy in treating multiple conditions including substance abuse (Carim-Todd et al., 2013), pain management (Cherkin et al., 2016; Nascimento et al., 2018), hypertension (Park et al., 2014), anxiety (Zeidan et al., 2014), depression (Kasala et al., 2014), and offering preventative effects in a healthy population (Wolsko et al., 2004; Barnes et al., 2008). However, the specific neural mechanisms of the practices’ broad range of benefits remain unknown.

Mindfulness meditation has received major attention in neuroscience research. It can be described as “non-judgmental attention to present-moment experiences” (Ospina et al., 2008). A proposed mechanism for the practice is that it works through a combination of enhanced self-regulation, including attention control, emotion regulation, and self-awareness. In general, there are two distinct regulation strategies (Chiesa et al., 2013). The first is “top-down,” where active reinterpretation of stimuli modifies emotional impact (Gross, 1998). The second strategy is “bottom-up,” where emotional regulation is achieved through direct modulation of brain regions associated with emotion-generation (Chambers et al., 2009; Westbrook et al., 2013).

Much of this evidence comes from functional and structural neuroimaging studies, typically employing magnetic resonance imaging (MRI). Structural studies often use voxel-based morphometry (VBM) to investigate changes in gray matter (GM; Fox et al., 2014). This well-established method entails a whole-brain structural analysis that examines local changes in gray matter volume (GMV). For a thorough review of morphometric studies of meditation, see Fox et al. (2014).

Morphometric studies of meditation have consistently found GM changes in regions related to the self-regulation mechanism mentioned above (attention control, emotion regulation, and self-awareness). These include the insula (involved in interoception, homeostatic awareness, and emotional awareness; Lazar et al., 2005; Hölzel et al., 2008; Luders et al., 2012), somatomotor cortices (tactile processing, conscious proprioception; Lazar et al., 2005; Grant et al., 2010; Luders et al., 2012; Kang et al., 2013), amygdala (fear, memory, decision-making, and detecting salient events; Pickut et al., 2013; Lu et al., 2014; Gotink et al., 2018), and posterior cingulate cortex (PCC; emotional salience and memory; Hölzel et al., 2011; Kang et al., 2013).

There are important questions that remain unanswered. For example, studies typically recruit experienced/expert adult meditators (Fox et al., 2014). Few studies are focusing on novice or meditation-naïve participants, and there are nearly no studies examining meditation’s morphometric effects in adolescents. This is an important knowledge gap because the adolescent brain is quite distinct from the adult brain and meditation effects may differ significantly (Giedd et al., 2015).

Adolescence is a crucial maturational period that begins at the onset of puberty (Dahl et al., 2018). In childhood, brain development is characterized by cortical gray matter growth. Cortical gray matter volume increases rapidly in the first few years of life and peaks in early childhood (Giedd et al., 1999; Shaw et al., 2008). In adolescence, however, this trend reverses. GMV decreases throughout adolescence towards young adulthood, with most regions following an inverted U-shaped trajectory (Shaw et al., 2006, 2008; Giedd et al., 2008). There are regional differences in the timing of this trend, with different structures maturing at different times and different rates (Blakemore, 2012; Wierenga et al., 2014; Narvacan et al., 2017). These unique developmental trajectories suggest that the findings from adult meditation studies may not translate to the adolescent population. Currently, only Friedel et al. (2015) have published structural findings in adolescents related to trait mindfulness. The authors found that trait mindfulness (measured at 19 years) was associated with less cortical thinning in the left anterior insula (AI) between mid- and late adolescence. While furthering our understanding of trait mindfulness in adolescents, the study did not employ any form of meditation training.

In the present study, we examined adolescents without previous meditation experience who underwent Training for Awareness, Resilience, and Action (TARA), a 12-week training program that incorporates elements from mindfulness meditation and yoga-based movements (Henje Blom et al., 2014, 2017). We compared participants’ gray matter volumes immediately before and after the training, as well as before and after a control period without any training.

## Materials and Methods

### Sample and Training

The study was approved by the Institutional Review Board (IRB) of the University of California, San Francisco, and all participants in the study provided written informed assent and their parent(s) or legal guardian(s) provided written informed consent following the Declaration of Helsinki. A community sample of 38 adolescent volunteers (16.48 ± 1.29 years, range 13.92–18.99 years, 24F) participated in this longitudinal neuroimaging study. Adolescent participants were recruited using IRB-approved flyers posted in the neighborhood. Exclusion criteria were any mental health conditions preventing effective group participation, such as active psychosis, severe anorexia nervosa, acute, and severe posttraumatic stress disorder, severe self-harm, suicidal ideation, or attempts in the past 3 months, or severe substance use disorder. Additionally, individuals with a diagnosis of intellectual disability or autism spectrum disorder were excluded. Adolescents undergoing current mindfulness training (e.g., mindfulness-based stress reduction, mindfulness-based cognitive therapy, or dialectical behavioral therapy) and/or an ongoing meditation and/or yoga practice of >20 min twice a week or more for the past 2 months were also excluded. Lastly, individuals with contraindications for MRI scans such as pregnancy, metallic implants, braces, or cardiac pacemakers were excluded. Given the relevance of TARA to conditions such as major depressive disorder (MDD), anxiety disorder, and attention deficit and hyperactivity disorder (ADHD; Henje Blom et al., 2014), we did not exclude participants with these disorders. Instead, we performed analyses with and without these participants. See Table 1 for specific demographic details.

**Table 1:** Demographic information of the participating sample.

	Primary (*n* = 38)	Control (*n* = 21)	Unmedicated (*n* = 32)
Mean age ± SD (years)	16.48 ± 1.29	16.59 ± 1.04	16.48 ± 1.33
Male;Female (count)	14;24	7;14	10;22
Psychiatric diagnoses	6 ADD/ADHD, 2 MDD, 1 GAD	5 ADD/ADHD	1 ADD, 1 GAD
On psychotropic medication	6	3	0

*Subjects were adolescent volunteers drawn from the community. Three VBM analyses were conducted: (1) primary, (2) control composed of subjects with an additional MRI scan 12 weeks before training, and (3) unmedicated subjects pre- and post-training. ADD/ADHD, attention deficit disorder/attention deficit hyperactivity disorder; MDD, major depressive disorder; GAD, generalized anxiety disorder; VBM, voxel-based morphometry*.

As part of the study, all participants underwent the TARA program (Henje Blom et al., 2014, 2017). TARA is a training program that incorporates elements from mindfulness meditation and yoga-based practices, which specifically promotes both bottom-up and top-down strategies to target depression and anxiety symptoms (Henje Blom et al., 2017). Subjects attended 90-min weekly sessions for 12 consecutive weeks. Two TARA-trained facilitators led the group in exercises such as guided breathing practices, yoga-based movement synchronized with breaths, and interoceptive/sensory awareness meditation practices such as body-scans. Sessions also included brief psychoeducational presentations and group discussions. Participants were instructed to continue practicing outside of the weekly session.

### MRI Data Acquisition

All 38 subjects underwent two MRI scans: one immediately before the TARA training (“pre”) and one immediately after the training ended (“post”). A subset of 21 subjects had received a third, control MRI scan. This additional scan was acquired 12 weeks before the training commenced. This subset was formed based on the time of study enrollment concerning the MRI and TARA schedule and not based on any other systematic difference between participants.

Each MRI scan was performed using a 3T General Electric MR750 MRI (Waukesha, WI) scanner. The scan included a standard T1-weighted (T1w) IR-SPGR sequence, with TR / TI / TE = 10.2 s / 450 ms / 4.2 s, flip angle = 15°, and 1 mm isotropic resolution. The ASSET acceleration factor was set to 2, with a total scan time of 3 min and 50 s.

### VBM Processing, Analysis, and Statistics

The optimized voxel-based morphometry was performed using FSL v5.0.8 tools (Good et al., 2001; Smith et al., 2004). The processing and analysis were not conducted blindly to timepoint. Non-brain tissue was removed from T1w images using *bet* (Smith, 2002). Each subject’s image was visually inspected for quality. Poorly extracted volumes were manually skull-stripped using a combination of *bet* and the *3dUnifize* and *3dSkullStrip* tools from the Analysis of Functional NeuroImages (AFNI version 18.1.18) suite (Cox, 1996). Images were segmented into gray matter, white matter, and cerebrospinal fluid (Zhang et al., 2001). The GM images were registered to the MNI152 brain at a resolution of 2 × 2 × 2 mm using affine (Jenkinson and Smith, 2001; Jenkinson et al., 2002) and non-linear (Andersson et al., 2007a,b) transforms. Thereafter, a study-specific template was created as part of the optimized VBM protocol implemented in FSL to which the GM images were non-linearly reregistered. The registered GM images were modulated by the Jacobian of the warp field to compensate for contraction/enlargement due to the non-linear transformation (Good et al., 2001). This obviated the need to correct for total intracranial volume (Scorzin et al., 2008) and permitted inference on local GM volume differences. Finally, smoothing with a Gaussian kernel [*σ* = 2 mm ≈ 4.7 mm full width at half maximum (FWHM) was performed].

The resulting data were subjected to voxel-wise permutation-based nonparametric methods (Nichols and Holmes, 2002) that corrected for multiple comparisons across space and incorporated threshold-free cluster enhancement (TFCE; Smith and Nichols, 2009). The tests used a repeated-measures paired *t*-test design that controlled for gender and included 5,000 permutations. This yielded two TFCE statistical maps, one for each contrast direction (pre > post or pre < post). Each map was thresholded at *p* = 0.025 (TFCE-corrected for family-wise errors) to account for the two contrast directions. The obtained regions were identified anatomically using the MNI Structural Atlas (Grabner et al., 2006).

To calculate the effect size, we extracted pre- and post-training GMV values for each participant in significant regions. GMV values were obtained from individuals’ modulated and smoothed GM images with *fslmeants*, using the significant cluster as a binary mask. Cohen’s *d* was calculated using a pooled standard deviation in R (v3.6.2; R Core Team, 2019) using the *effectsize* package.

To assess the potential relationship of age to GMV change, we conducted a bivariate Pearson correlation analysis between participants’ ages at the beginning of the training and their GMV changes across the training, also in R.

### Control and Unmedicated Analysis

A second control, VBM analysis was conducted with the subset of participants who received the control scan (*n* = 21, 16.70 ± 1.07 years, 14F). This analysis examined structural changes between the control and pre-training timepoints, and it provided a comparative window of subjects’ typical development that incorporated background factors such as biological development and environmental stressors such as school.

A third, VBM analysis was conducted to compare medicated and unmedicated subjects (*n* = 32, 16.48 ± 1.33 years, 22F). This analysis examined unmedicated subjects’ changes between pre- and post-training. Both the control and unmedicated analyses used the same processing and statistical tests as the primary VBM analysis.

## Results

### Data Acquisition

MRI scans were well-tolerated by all participants. Scans were read by a board-certified neuroradiologist. No one had any abnormality and was excluded from the analysis.

### Primary VBM Results

**Figure 1:**
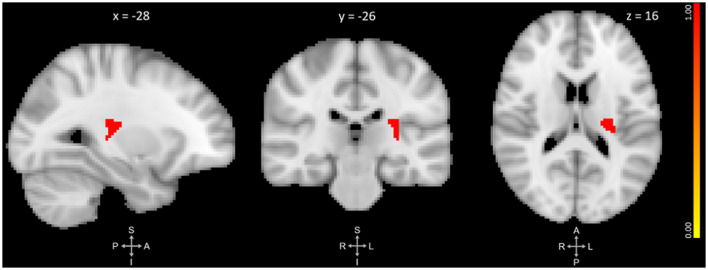
Three-plane view of the region showing significant gray matter (GM) volume decrease after meditation training. The cluster showed the most significant change at the left posterior insula [PI; *p* ≤ 0.025, threshold-free cluster enhancement (TFCE)-corrected for family-wise errors]. The cluster also overlapped with the left thalamus and left putamen. The cluster is overlaid over the MNI 2 mm brain template, and coordinates are in MNI standard space. The right-side color bar corresponds to the cluster’s significance (1-p).

The primary VBM analysis identified one cluster that showed significant GMV reduction between pre- and post-training. The cluster extended medially into the left thalamus and left putamen with its peak in the left posterior insula (*p* = 0.019, *d* = 0.47). No regions showed significant GMV increase. See Figure 1 and Table 2 for results.

**Table 2:** Primary VBM results of posterior insula gray matter volume decrease pre- to post-training.

Region	Side	Size (mm^3^)	x	y	z	Sig. (−)	Sig. (+)
Posterior Insula	L	216	−28	−26	16	0.019	1

*Peak significance is reported, at the α = 0.025 level (TFCE-corrected for family-wise errors). The cluster’s peak coordinates are in MNI standard space. L, left; Sig., significance; (−), decreasing direction, (+), increasing direction*.

There was no significant association between participants’ age and GMV changes during the training, *r*_(36)_ = 0.0197, *p* = 0.907.

### Control and Medication Effect Analysis

There were no regions of significant GMV increase or decrease in the control VBM analysis. The VBM analysis comparing medicated with unmedicated subjects showed a trending decrease of GMV at the left posterior insula peak from the primary analysis (*p* = 0.07). See Table 3 for details.

**Table 3:** Results of the control and unmedicated VBM analyses.

Analysis	*n*	Side	x	y	z	Sig. (−)	Sig. (+)
Control	21	L	−28	−26	16	1	0.168
Unmedicated	32	L	−28	−26	16	0.07	1

*The VBM analysis was repeated with a control subset (*n* = 21) and an unmedicated subset (*n* = 32). In both analyses, no regions showed statistically significant differences in GMV between timepoints. The L-posterior insula peak coordinates yielded trending decreases in the unmedicated analysis. L, left; Sig., significance; (−), decreasing direction; (+), increasing direction*.

## Discussion

### Overview

Our primary VBM analysis identified a left posterior insula cluster showing significant GMV decrease after meditation training. Insular changes have been reported in previous meditation studies and this region is key for physical and emotional awareness. The cluster also overlapped into the left thalamus and left putamen. The following sections will discuss the regions involved and the potential mechanism of change.

#### Insula

Structural differences in the insula (insular cortex) are one of the most well-replicated findings amongst morphometric studies of meditation (Lazar et al., 2005; Hölzel et al., 2008; Luders et al., 2012; Murakami et al., 2012; Friedel et al., 2015). The insula is crucial for interoception, that is, one’s sense of one’s internal physiological state (Craig, 2002). It is thought that the insula accomplishes this by receiving homeostatic stimuli and sensations and refining them into higher-level awareness (Craig, 2009). It has three cytoarchitecturally distinct components that work together to carry out this function: the granula posterior insula (PI), the dysgranular middle insula (MidI), and the agranular anterior insula (AI; Gu et al., 2013).

The posterior insula provides the primary interoceptive representation of one’s physiological condition. Bodily stimuli, such as temperature, pain, itch, and respiration, are brought to the posterior insula *via* lamina I spinothalamic neurons (Craig, 2002; Brooks, 2006; Craig, 2009; Strigo and Craig, 2016). These are sent forward to the AI and during this posterior-to-anterior transition through the MidI, the viscero-somatic information is refined. Evidence suggests that the MidI integrates interoceptive stimuli with other neural inputs (Craig, 2011). This helps create a salient representation of an individual’s homeostatic features. For example, the MidI was associated with both taste perception (Dalenberg et al., 2015) and the coding of a food’s pleasantness (van Rijn et al., 2018). The AI assigns emotional relevance to the incoming homeostatic stimuli, thus underlying emotional awareness (Craig, 2009; Fox et al., 2018). Functionally, the AI is a key node of the Salience Network (SN), the intrinsic functional network that identifies biologically and cognitively relevant events to guide behavior (Seeley et al., 2007; Menon and Uddin, 2010; Menon, 2011).

Our results were in the posterior insula, which differs from previous insular findings which are typically located in the anterior insula (Fox et al., 2014). This distinction is important because the PI and MidI have been associated with objective ratings of stimuli, whereas the AI has been associated with their subjective ratings (Craig, 2002, 2011; Singer et al., 2004). For example, objective changes in temperature stimuli were associated with the PI, but subjective evaluation of the same stimuli was associated with the AI (Craig et al., 2000; Kong et al., 2006; Craig, 2009). The posterior findings may reflect the TARA training’s emphasis on interoceptive physical awareness practices, such as breathing exercises and “body-scan” meditations.

Prior studies with significant insula findings utilized meditation paradigms which explicitly focused on body awareness, posture, breathing, and other physical sensations (Lazar et al., 2005; Hölzel et al., 2008; Kang et al., 2013; Fox et al., 2014; Tang et al., 2015). Thus, the anterior-posterior position of insula findings may correspond to the type of meditation practice applied.

#### Thalamus

The significant cluster also overlapped with the left thalamus. This is important because of the thalamus’ function and its relationship with the insula. The thalamus is a large collection of nuclei that relays information to the cortex, and it is particularly crucial for the transfer of sensorimotor information (Vertes et al., 2015). The GMV changes in our VBM analysis could reflect this specific function, as participants repeatedly practiced exercises involving sensory awareness and physical movement. Additionally, the thalamus projects lamina I spinothalamic nuclei into the posterior insula (Craig, 2002, 2009). Significant GM changes in a cluster containing both the thalamus and the posterior insula further support an awareness-based mechanism of meditation’s effects.

Previous studies have implicated the thalamus in meditation practice, but these used other imaging modalities such as single-photon emission computed tomography (SPECT; Newberg et al., 2001) and functional MRI (fMRI; Lutz et al., 2008). Luders et al. were the only group to have found GM changes in the thalamus associated with meditation. They observed larger right thalamic GMV in long-term meditators (Luders et al., 2009), in contrast to our VBM results of GMV decreases. This discrepancy could be due to differences in the participants’ ages (adults vs. adolescents) and meditation experience (experienced meditators vs. novices).

#### Putamen

The left PI cluster also overlapped into the left putamen. The putamen is a subcortical structure and is part of the dorsal striatum. It is involved in the refinement and control of motor movement (Balleine et al., 2007). Additionally, it is associated with the reinforcement of learning (Viñas-Guasch and Wu, 2017), reward-related behavior (Packard and Knowlton, 2002), and emotions (Brooks, 2006; Lanciego et al., 2012). Amongst its many connections, the putamen is part of multiple corticostriatal loops and has projections into the thalamus.

Our findings in this region can potentially be explained by the training’s focus on physical movements. In addition to the awareness practices previously mentioned, all sessions included yoga-based movement exercises. Here, subjects initially learned a few movements. These previously-learned movements were repeated each session while new sequences and poses were added. One of the key components of the motor circuit is a corticostriatal projection from the putamen (Lanciego et al., 2012). GMV decreases in the putamen could be due to repeated engagement of motor circuitry to learn these coordinated motor movements.

Multiple fMRI studies have reported changes in putamen with meditation (Baerentsen et al., 2010; Ding et al., 2015; Kirk and Montague, 2015; Hernández et al., 2018). The only morphometric structural findings are reported by Pagnoni and Cekic (2007). They also found GMV changes in the left putamen, although their finding was that controls, not practitioners, showed GMV decline. The authors suggest that meditation provided a neuroprotective effect that halted the decline in their adult practitioners. As our sample consisted of adolescents, meditation’s neuroprotective effect could be manifesting itself in some other manner, such as enhancing the brain’s development.

### Gray Matter Volume Decrease

The most surprising result of our VBM analysis was the direction of GMV change. Subjects showed significant decreases in GMV after the meditation training. This contrasts with most structural MRI studies of meditation, in which GM measures increase with practice (Fox et al., 2014). However, findings of morphometric decrease (or those in which changes in controls were larger than in meditators), are not without precedent (Kang et al., 2013; Gotink et al., 2018).

Structural MRI studies of meditation have focused on adult samples. The discrepancy between our results and previous studies’ results could be partly explained by the younger age range of our sample (16.48 ± 1.29 years). It is important to take into consideration the differences between the adolescent brain and the adult brain. Several studies have shown that GM morphometric measures (volume, thickness, etc.) follow an inverted-U shaped trajectory, peaking in early adolescence and then declining towards young adulthood (Giedd et al., 1999; Shaw et al., 2006, 2008; Narvacan et al., 2017). Developmental evidence also indicates that the relationship between GM structure and functional abilities undergoes significant changes throughout the lifespan. A study by Schnack et al. (2015) found that in childhood, thinner cortices were associated with higher intelligence. However, this relationship reversed in young adulthood (~21 years) so strongly that in adulthood (42 years), thicker cortices were associated with higher intelligence (Schnack et al., 2015). Thus, the decreased GMV findings in our study could reflect a regionally-specific increase in maturation during the 12 weeks of TARA-training. While possibly the observed GMV decreases were driven solely by maturation, the lack of GMV decreases during the control period render this unlikely.

In mid- to late adolescence, the brain undergoes global stabilization and refinement that ultimately enhance functioning and efficiency (Dahl et al., 2018). One mechanism is through activity-dependent stabilization and synaptic pruning (Changeux and Danchin, 1976). Functionally, this process is thought to improve neuronal stabilization by solidifying experience-dependent learning, reducing spine density, and improving connection efficiency (Anderson et al., 1995; Dahl et al., 2018). This process is a key component of adolescent maturation. Animal studies have observed higher rates of dendritic spine formation and elimination within the adolescent brain compared to adults (Drzewiecki et al., 2016). Additionally, pruning has been hypothesized to account for the GMV decreases observed in late adolescence (Sowell et al., 2001; Gogtay et al., 2004; Blakemore, 2012).

We speculate that our decreased GMV findings could be explained through this developmental maturation context. Meditation practices could have bolstered the maturation of neuronal connections in physical awareness regions (posterior insula) and regions necessary to carry out and learn such techniques (thalamus and putamen). This refinement may have reduced transient spine formation within the regions, ultimately leading to GMV volume decrease. Though speculative, this mechanism can explain the differences between our GMV findings and those from the meditation studies on adults.

### Limitations

This study’s findings should be interpreted in light of its limitations. First, the study was not randomized. However, the study design included a within-subject control: a subset of the adolescent subjects received an additional scan before the pre-training timepoint, allowing us to compare findings to a period where subjects had no training. VBM analysis yielded no significant change during the control period, supporting the conclusion that the primary findings were due to training-related practices. This controlled within-subject design can be potentially more powerful than a between-subject design, as within-subject variability is much smaller than between-subject variability (Poldrack, 2000). Second, the study sample was heterogeneous: nine subjects had a psychiatric diagnosis (six with ADD/ADHD, two with MDD, and one with GAD). Six subjects were receiving medication and due to this small number and the different types of medications, we could not thoroughly examine the effects of medication. We performed an additional VBM analysis excluding participants taking medication to compare overall differences and the findings in the left posterior insula were preserved at a trending level, supporting the primary findings. Third, TARA training is an amalgamation of different practices (i.e., meditation, breathing exercises, and yoga-based practices). Currently, the individual contribution of each component practice is unknown. Future studies are needed to disentangle whether and how much each component contributes to the overall structural effect. Fourth, VBM is a method based on T1-weighted MR images and as such, any tissue property that affects T1 relaxation times (e.g., cell density, cell size, myelination) could affect a voxel’s intensity (Zatorre et al., 2012). For example, cerebral blood flow changes (*via* caffeine ingestion or sensorimotor task), can cause apparent tissue changes in VBM analysis (Ge et al., 2017). It is important to interpret VBM findings with this limitation in mind. Finally, we did not assess behavioral changes, such as measures of anxiety and depression psychopathology. Additional research is needed to study whether these morphometric changes are reflected in behavioral changes.

## Conclusion

Overall, we found significant gray matter volume decreases in adolescents participating in a 12-week training program involving meditation, breathing exercises, and yoga-based movements. The GMV changes were centered on the left posterior insula, a region crucial for interoceptive awareness. Whereas most structural neuroimaging studies have found increased GM measures with meditation practice, we found the reverse: participation in meditation training was associated with structural decreases. The discrepancy could be explained by the unique maturational stage of adolescence and its structural brain changes. These findings help further our understanding of meditation’s effects in the understudied adolescent brain.

## Data Availability Statement

The datasets generated for this study are available on request to the corresponding author.

## Ethics Statement

The studies involving human participants were reviewed and approved by Institutional Review Board (IRB) of the University of California, San Francisco. Written informed consent to participate in this study was provided by the participants’ legal guardian/next of kin.

## Author Contributions

JY, EH, TY, DX, and OT designed the study. JY, EH, DX, and OT collected the data. LS read MRI scans for incidental findings. JY, CC, and OT processed and analyzed the data. JY wrote the manuscript. CC, EH, LS, TY, DX, and OT co-wrote the manuscript.

## Conflict of Interest

The authors declare that the research was conducted in the absence of any commercial or financial relationships that could be construed as a potential conflict of interest.
